# ‘Brought in Dead’: Post-Mortem Glimpses of the Early ‘Heroin Epidemic’ in Ireland, 1971–1983

**DOI:** 10.1093/shm/hkae094

**Published:** 2025-06-29

**Authors:** Oisín Wall

**Affiliations:** School of History & Radical Humanities Laboratory, University College Cork, Cork, Ireland

**Keywords:** Drugs History, Opiates, Barbiturates, Ireland, Death

## Abstract

This article explores the formation of Ireland’s first ‘hard drug’ culture. To do this, it uses the coroners’ reports on drug-related deaths in Dublin between 1971, when the first overdose by a regular user was recorded, and 1983, when the first Irish ‘heroin epidemic’ peaked. Through these reports, the article constructs a macro-view of the demographics involved in ‘hard drug’ use and the changing trends within the subculture. It contrasts this overview with the lived experience of the drug culture by developing a series of micro-histories of specific people who used drugs during this period, which both illustrate and counterpoint the statistical trends. In doing so, it demystifies the ‘hard drug’ culture and reinserts it into the history of Irish everyday life.

## Introduction

By 1983 people were confidently referring to Dublin as the ‘heroin capital of Europe’.^1^ This may seem hyperbolic, given the high rates of heroin use elsewhere on the continent, but it represents a very real fear of the rapid expansion of Dublin’s drug culture. Unlike other cities which had serious heroin cultures, just 15 years earlier, in 1968, there was no evidence of a ‘hard drug’ culture anywhere in Ireland. However, by 1983, one in 10 of 15- to 24-year-olds in parts of Dublin were using heroin.^2^ The rapid expansion of the Irish drug culture is often attributed to singular exceptional forces like the proliferation of heroin after the Iranian revolution in 1978 or the decision by a particular family-gang to start importing drugs to Dublin at the beginning of the 1980s.^3^ Such explanations paint the ‘hard drug’ scene as Other—an international criminal demimonde made comfortably alien from the reader’s reality by its susceptibility to these exceptional forces.^4^ This article will challenge the singular-cause perception, and complicate the history of the drug scene in Ireland: firstly, by demonstrating that, while the formalisation of the drug supply did lead to changes, the ‘hard drug’ culture was already well established by the beginning of the 1970s; and secondly, by illustrating the normality of the drug scene and its existence within the continuum of Irish everyday life.

To achieve this, the article will use the case files of the Dublin City Coroner’s Court. As Ciara Breathnach recently argued, through their incorporation of expert and witness testimony, coroners’ files give us access to histories from both above and below.^5^ It is the role of the coroner to look into any deaths which are violent, unnatural or sudden and unexplained. Given the unexpected nature of these deaths, the moment of mortality is not often witnessed and so, more often than not, the files are made up of statements about the deceased’s final hours or days and observations about whether their actions were normal or in keeping with their usual routine. The result is that, far from being about death, many of the documents contained in coroners’ files are really glimpses of everyday life. In these files, we find descriptions of ordinary days: who people ‘palled around’ with, where they ate their dinner, and their role in family or social life, as well as how and where they used their drugs. As such, by examining the Dublin City Coroner’s files about drug-related deaths it is possible to ‘feel right round all the sides’ of the drug scene in 1970s and early-1980s Ireland.^6^ The drug culture of the 1970s in particular remains a deeply under-researched area of Irish cultural and social history, with little written on the topic. Last year James Grannell and I published an article, giving an overview of twentieth-century public discourses around heroin use which touched on the period.^7^ In the early 2000s, Jo Murphy-Lawless published an account of women’s resistance to the drug-use in Dublin, while Julie O’Toole and André Lyder have published biographies about their time using drugs and involvement with the ‘pushers out’ movement, respectively.^8^ However, these focused on the 1980s and 1990s. There have also been a handful of policy and medical histories that touched on the development of the heroin epidemic which also focused on the 1980s.^9^ However, this article will show that the 1970s was an important and formative period in the Irish drug culture.

I use the image of feeling around the sides of the subculture advisedly because, as in Abbott’s *Flatland*, these files give us access to only the extreme edges of Irish drug culture. Through the files, we mainly see glimpses of the lives of people at the disorganised or disintegrating edges of the culture—by this I largely mean people new to drug-use who do not yet know what they are doing and how to use safely, or people who have been using for many years and due to changing circumstances are becoming increasingly reckless in their use. What is largely obscured, or only hinted at, by this approach is the vast interior of the culture in which people held down jobs and family lives while using drugs regularly or semi-regularly. I hope to explore these aspects of the culture in other publications, but for now, we will begin by mapping the edges.

## Definitions, Methodology and Limitations

Before we begin mapping the ‘hard drug scene, however, it would be appropriate to discuss what we mean by this term. ‘Hard drugs’ was a widely used term in late 20th-century Ireland. A frequency analysis in three leading newspapers (*Irish Independent*, *Irish Times* and *Irish Press*) found that it occurred in 398 articles between 1971 and 1983. However, it was not a distinct set of drugs, but rather a vague set of illicitly obtained drugs that were believed to cause serious long-term effects for the user, including addiction and death. Although this was not a firm category of specific drugs, there were fixed points of reference. Opiates like heroin were always included, cocaine was sometimes mentioned, while illicitly used pharmaceuticals, including barbiturates (prescribed as sedatives) and anti-cholinergic (prescribed to treat spasms), were among the most common features.

The article will use the terms ‘hard drug scene’, ‘drug culture’ and subculture interchangeably. The first two were commonly used terms at the time, the third is intended in a more technical sense. For a working definition of subculture, the article draws on that offered by Stuart Hall et al in *Resistance Through Rituals*—‘Culture is the way the social relations of a group are structured and shaped: but it is also the way those shapes are experienced, understood and interpreted.’^10^ Within this frame subcultures are bound to their ‘parent’ culture, sharing many of the same shapes of social relations and ‘maps of meaning’ that guide the interpretation of these shapes.^11^ However, they are also at variance with the ‘parent’ culture in the ‘activities, values,[…] uses of material artifacts, territorial spaces, etc.’ around which they have coalesced.^12^ Over the course of this article, we will see many of the points at which the subculture, which coalesced around ‘hard drugs’, connected with the culture which surrounded it, but we will also see the growing variance between this ‘hard drug scene’ and its ‘parent’ culture.

For this article, I have assessed the files of every death that the coroner recorded as drug-related between 1971, when the first confirmed deaths from recreational opiate use were confirmed, and 1983. The article ends in 1983, because, as I have discussed elsewhere, that was year that the Irish heroin epidemic peaked and so the year marks the end of a period of rapid subcultural growth.^13^ Two other factors caused significant shifts in the subculture at the same time. The Pushers Out movement, which targeted people perceived to be selling drugs in working-class communities, began to take shape in 1982 in the Hardwicke Street Flats in the North inner city, and by 1983, it was developing momentum and beginning to have a significant impact on the supply of hard drugs across the city, and the lives of people who used them.^14^ Also in 1982 the first case of AIDS was identified in Ireland, and in 1983 this entered the public discourse through newspaper articles like ‘Killer Disease is Here’.^15^ AIDS, and the discourse that surrounded it, would go on to have a transformative influence on drug-use, and particularly injecting culture, in Ireland through the mid- and late-1980s. This makes 1983 a natural end-point for the study, as well as a provocation for future research.

To narrow the study, I have focused on the Dublin City Coroner. It should be noted that the County Coroner’s jurisdiction covered much of the Northern and Southern suburbs, including Dun Laoghaire, where there were many drug-related deaths. As such the study captures a significant sample, but not all of the drug-related deaths in the Dublin area. Moreover, I have only examined cases where ‘drugs’, ‘overdose’ or any related words were recorded in the Coroners’ Register. During this search, I cast a wide net for related words and assessed several hundred case files, finding that drug-related deaths seem to have always been recorded in the register with some allusion to drugs. In light of this, I am confident that this parameter has not excluded too many relevant cases. I have also ruled-out cases that the coroner deemed to be suicide, where there was not an apparent prior history of drug-use. This was quite a small group because suicide was criminalised in Ireland and coroners were restricted from implicating the deceased in a crime.^16^ I have also excluded accidental overdoses of prescribed medication where there was no suggestion that the medication was being consciously over used. This is a large group and the one where relevant cases are most likely to have been excluded. The large amounts of prescribed barbiturates and the prevalence of heavy social drinking meant that there was a large number of people who legitimately fell into this group, but it is possible that people whose drug-use was emergent, secretive or otherwise unrecorded, may have been miscategorised here. The breadth of this group raises an interesting question about the impact of gender and class biases on coroners’ recorded findings, which certainly deserves further study.

The remaining group, which is the focus of this study, amounts to 38 cases, a surprisingly small figure given the ‘epidemic’ discourse of the period.^17^ Intriguingly, the deaths fall into two distinct clusters: the first seventeen deaths occurred in the 6 years between 1971 and 1976, and the second 21 deaths occurred in the 5 years between 1979 and 1983. Curiously, these two periods were separated by a 2-year period, 1977–8, in which there were no recorded drug-related deaths.

All coroners’ reports are public documents and many of the coroner’s court cases were reported in the press, often including the full names and addresses of the deceased and their families. Nonetheless, it may be upsetting to surviving family and friends to have their loved ones’ names republished now and in the context of this article. In light of this consideration, I have randomly assigned names (using lists of common Dublin names) to anyone mentioned in this article who was not acting in an official capacity, like gardaí, judges and coroners.

Over the course of this article, I will use a statistical analysis to begin to plot the shape of drug culture in this period, exploring the commonalities between these two periods, as well as the things that distinguished them. While the 1971–6 and 1979–83 periods were markedly different, they also share important commonalities. These common trends are important because they demonstrate that what we are looking at are two periods of the same culture, rather than two distinct cultures. These statistical analyses are extremely useful but can obscure the ordinariness of the lived experience of the drug culture. To counteract this, I have included stories of individuals with each analysis, which illustrate or counterpoint the statistical trend. However, before we start examining these tendencies, it is important to interrogate the nature of the material through which we are looking and ask how it distorts the image that we see.

### Distortions: The Case of Derek Connolly^18^

Derek Connolly died in 1981, aged just 15 years. Born in England to an Irish couple, the family moved back to Dublin when Derek was 6 years old. They settled in a flat on Sillogue Road in Ballymun, a recently built suburb in North County Dublin. Derek had a difficult time in school and ultimately left at the age of 13. After this, his father, a painter-decorator, would often take Derek to work with him. After Derek left school his father began to suspect that he was using drugs. This was confirmed in June 1981, about a month before Derek’s death when he admitted to his father that he was taking barbiturates. After this, he tried to get off the drugs, initially by staying home and avoiding his friends, and then by arranging to be admitted to the Jervis Street Hospital’s drug unit. However, he did not last long in the unit and discharged himself after just 4 days.

Less than a week after leaving hospital, on 1 July, he told his parents that he was going out to a disco, but instead met up with his friend John Murphy (17 years old) and took ‘a good lot of drugs’.^19^ At around midnight the pair decided to break into a chemist’s shop, in Whitechurch, just South of Ballymun. However, a passing Garda patrol, having spotted the window that the boys broke to get in, arrested them in the middle of the robbery. The arresting officer described them as ‘two frightened youths… [who] offered no resistance and came quietly’.^20^ They were taken to Whitehall Garda Station where a Detective Garda Daly from the neighbouring Ballymun Station interrogated them about a series of burglaries in local schools. Derek, who was still visibly stoned, initially denied the accusation that he was involved with the school robberies, but later made a statement admitting to one of them.^21^ Derek was informed that he should call to the Ballymun Garda Station to be charged with the School burglary later in the month then, at 4 am, both he and John were charged with the chemist break-in and told that they were to appear at the Kilmainham Courthouse later that day (2 July). John recalled:

I don’t remember being charged with breaking into the shop or getting a copy of the charge sheet, I have a recollection of Detective Daly coming to Whitehall and into the room I was in. I don’t remember how I got home that morning… We did not go to the court on Thursday because we did not know or we were not sure we were supposed to. We had a lot of drugs taken the day before.^22^

Derek got home at around 5 am. He did not tell anyone in his family that he had been arrested or spent the night in the Garda Station. When his mother suggested that he had been with John, whom she believed was a bad influence, Derek denied it vehemently. That morning his father took him to work with him, but Derek was exhausted and coming down, so he left at 2 pm and went home.

The next day, Friday 3 July, Derek’s mother recalled finding John and another of Derek’s close friends, Anne Whelan (16 years old), behind the lifts in her flat block:

He [John] was very much under the influence of drugs. He was sniffing lighter gas and had a jar of capsules with him, I took the jar off him. He tried to get the jar off me but I smashed them in the grating. I then went up to get a bucket of water to wash them away, when I came down with the bucket of water John Murphy was taking the capsules up from muck. I pushed him aside and washed them away.^23^

Curiously, this event is not featured in either John or Anne’s recollection of the day. In the afternoon, Derek and John went to Stephen’s Green, a city-centre park, with a bottle of Actifed (a pseudoephedrine-based cough syrup, used recreationally as a stimulant). While they were there, a man sold them 11 tablets of ‘Dike’ (Diconal, an opioid with a similar strength to heroin) for £38.^24^ They each took two tablets, then got a syringe from Jervis Street Hospital and went back to Ballymun. In a green space at the back of John’s housing estate, they crushed and injected another two tablets each.^25^ John could not recall much of what happened after this, but Anne found them in John’s house not long later. She recalled that they were both ‘stoned’ when she arrived and that neither Derek nor John would tell her what they had taken.^26^ After a while the three friends made their way to the shed beside St Pappin’s Church, discarding some little green tablets on the way, which John declared to be ‘no good’. In the shed, the two boys took out their drugs, but Anne told them: ‘not to be messing with needles in front of me, as I hate needles’.^27^ Instead of using them in front of her, they went to the Ballymun Shopping Centre and the boys told her that they were going into the toilets for a minute. They came out half an hour later having taken the remaining Diconal tablets and were ‘completely stoned’.^28^ Anne went to one of the shops and the two boys sat on a bin outside where Derek kept falling asleep. John went home and a little later Anne found Derek unconscious in the entrance hall of his own flat block. She woke him, and he said he was going to have a bath and go to bed.^29^ When Derek’s mother came home at 9.30 pm she found him getting ready for bed and ‘he seemed alright’.^30^ When she tried to wake him, 12 hours later, she found him dead.

The death of Derek Connolly is, in many ways, typical of coroner’s reports of drug-related deaths in the early 1980s. What makes this case stand out, however, is that it reveals the power relations that hide in this archive. On the face of it, the coroner’s reports come across as relatively dispassionate accounts of ordinary, albeit tragic, events. The Connolly file, however, is between two and four times longer than most comparable cases. This is because eight separate gardaí gave statements, and many of Derek’s family and friends gave multiple statements, which was highly unusual. The reason for this extra level of paperwork was that after Derek’s death, his family started to ask difficult questions about his treatment by the Gardaí in his final days. Here, the facade of the dispassionate catalogue of facts begins to crack.

When his family found Derek’s body there were dark marks like bruises on his face, left hand and on the left of his torso. When his father asked the Gardaí about why his son was ‘black and blue’ the Sergeant told him that dead bodies ‘generally turned out that way’.^31^ A little later his sister, Mary Ellen Connolly, who also saw the marks, asked Anne about them. Anne told her about Derek and John’s arrest and said that while in custody Derek had been beaten up by the Gardaí and had shown her the bruises. She said John had also been beaten up but not as badly. Anne later gave a statement to that effect in Ballymun Garda Station, saying that Derek said he had been beaten with batons on his way into Whitehall Garda Station. She told the Gardaí that she had not told anybody up until that point because the Connolly family were not talking to her and blamed her for the death of their son.^32^ Derek’s father then engaged Dudley Potter, a prominent civil rights solicitor who often represented activist organisations like the Prisoners’ Rights Organisation as well as people making accusations of Garda brutality.

In light of this accusation, John slightly revised his statement to say that when he was being arrested in the chemist’s shop a Garda had hit him on the hand with a baton. However, he maintained that he had no memory of either himself or Derek being beaten in the Garda station.^33^ He restated this at the inquest, though confirmed that he remembered seeing Derek showing Anne his bruised leg.^34^ Inspector James Connell carried out a preliminary investigation of the allegations. In the course of the investigation, he said that John gave a verbal statement that ‘neither of them was beaten or ill-treated’, and recommended that the inquest should continue as planned. In the course of the report, however, he sought to discredit both John and Anne:

[John Murphy] also stated that he was aware that [Anne Whelan] (whose statement is in the original file) had made the rumour that they were assaulted at Whitehall Station. Both [Murphy] and [Whelan] have since been taken to hospital on more than one occasion under the influence of drugs.^35^

When the inquest was held on 4 December, Anne responded to this, amending her statement to say that she did not have a drug problem, although she had once been taken to hospital ‘suffering from drugs’.^36^ The doctor who performed the post-mortem, Dr Niall Gallagher, told the inquest that there were no bruises on the body, but that there was a common discolouration of the skin that occurs when blood pools at the lowest part of a corpse, called post-mortem lividity. He went on to say that this was often confused with bruising by lay people.^37^ The Coroner found that the death was caused by ‘respiratory depression due to an overdose of drugs’ and classified the death as ‘accidental’.^38^ When the verdict was read Derek’s father stormed out of the court in protest that the alleged Garda violence had not been taken into account.^39^

The Connolly file stands as a reminder that the coroner’s reports are not dispassionate accounts of deaths, but an official collection of statements taken by Gardaí and entered into the public record. Indeed, the written statements often form the basis for witnesses being called to give evidence in the public forum of the coroner’s court, where their answers may be reported by the press. All of this shapes the nature of the account that is recorded—incriminating or embarrassing details are often omitted; names of suppliers, friends or associates are conveniently forgotten and official improprieties go unrecorded. This file emphasises for us the importance of approaching the coroner’s archive with a degree of suspicion. Were Derek and John beaten by the Gardaí? Were the marks bruises or lividity? Did John deny any memory of violence because he still had charges pending against him? Was he worried that he would be extra-judicially punished by the colleagues of any Gardaí he named? Did Anne, or even Derek, invent or exaggerate the story of violence for personal reasons? As readers, we must live with these uncomfortable ambiguities, and we must allow them to haunt our reading of every case that follows.

### Who Was Dying?

**Fig. 1:**
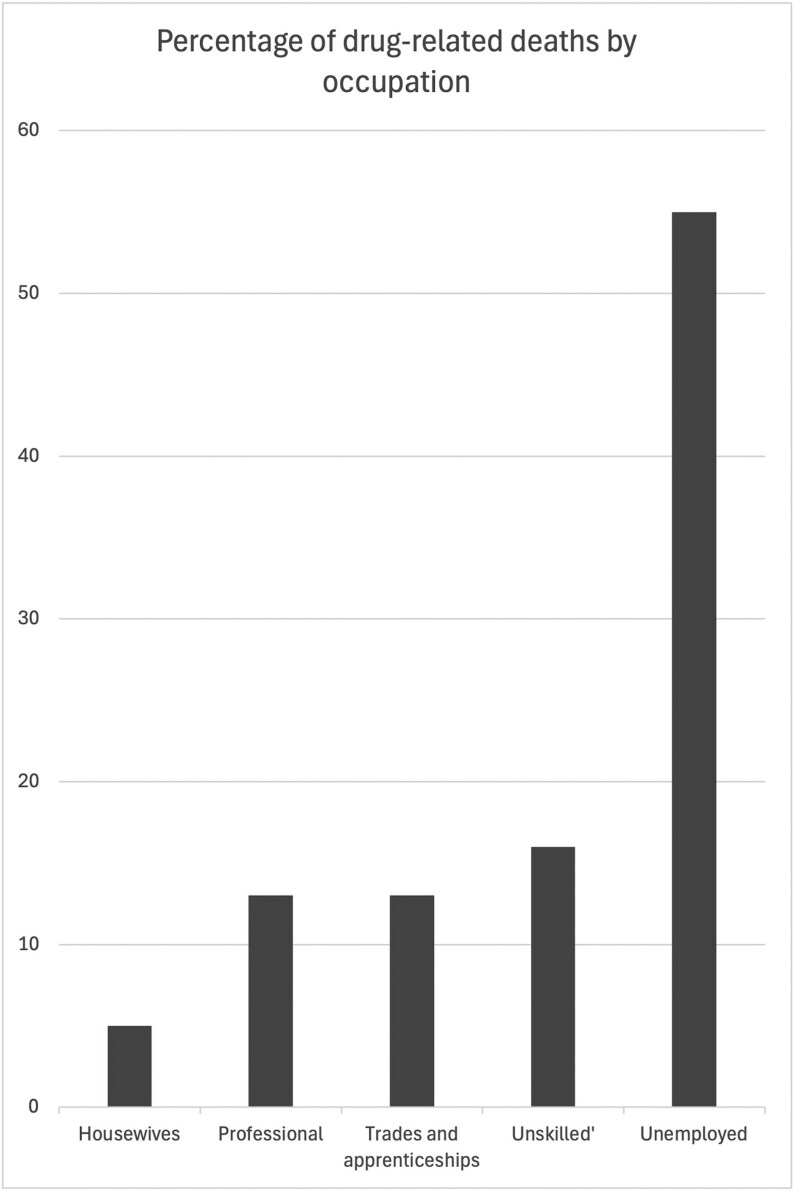
Percentage of drug-related deaths by occupation, 1971–83

While each case in the coroner’s archive represents a markedly different personal tragedy, taken as a whole they present a remarkably coherent picture. In this section, we will examine the class, gender and age profiles of the people represented by these files. Of all of the cases assessed for this study, only five (13 per cent) involved the death of people from middle-class or professional backgrounds (a teacher, a university student, a nun and a man with ‘independent means’). Two (5 per cent) were housewives whose marriages had ended, both seem to have been working class and in one case the woman’s ex-husband is recorded as being unemployed. Another five (13 per cent) involved people with trades or apprenticeships (two tailors, an upholsterer, a carpenter and an electrician). Six (16 per cent) were engaged in what have traditionally been considered ‘unskilled’ labour (a petrol pump attendant and five ‘labourers’). Finally, 21 (55 per cent) were recorded as being unemployed. I combined this information about occupations, with, where it was recorded, their parent’s occupations and the addresses of their family homes.^40^ From this, we can see that not only were the vast majority (up to 87 per cent) of people who died from drug-related causes from working-class backgrounds, but this number skews heavily towards the lower socio-economic end of the working class with unemployed people and their dependents or ‘unskilled’ labourers accounting for three-quarters of deaths across the period (Figure 1).

The gender and age profile was also extremely homogenous. 34 (89 per cent) of the people who died were men or boys. Interestingly, the profile of the men and women who died is starkly different. Across this whole period, only four women were recorded as dying from drug-related causes, they ranged in age from 33 to 63 and had an average age of 48. Two were from a professional background—a teacher and a nun, and the other two were the housewives mentioned above. All four died from overdoses taken at home, alone. Moreover, although each of them struggled with substance dependence there is no evidence that they were part of a social drug culture—none were known to the gardaí, there is no evidence that they had ever used with other people, and none of their friends or family who gave evidence at the inquest openly used drugs or seemed to know anything about the scene. The small number of cases in which women died, combined with their relative heterogeneity make it difficult to draw any convincing conclusions other than that women tended to die alone and at home. Even this minimal conclusion is more vague than it sounds, as ‘home’ here signifies several markedly different kinds of spaces, from temporarily rented flats that nobody ever visited to permanent suburban homes which daily hosted friends and family, to a bedroom in a convent with the communal lifestyle that that implies.

**Fig. 2:**
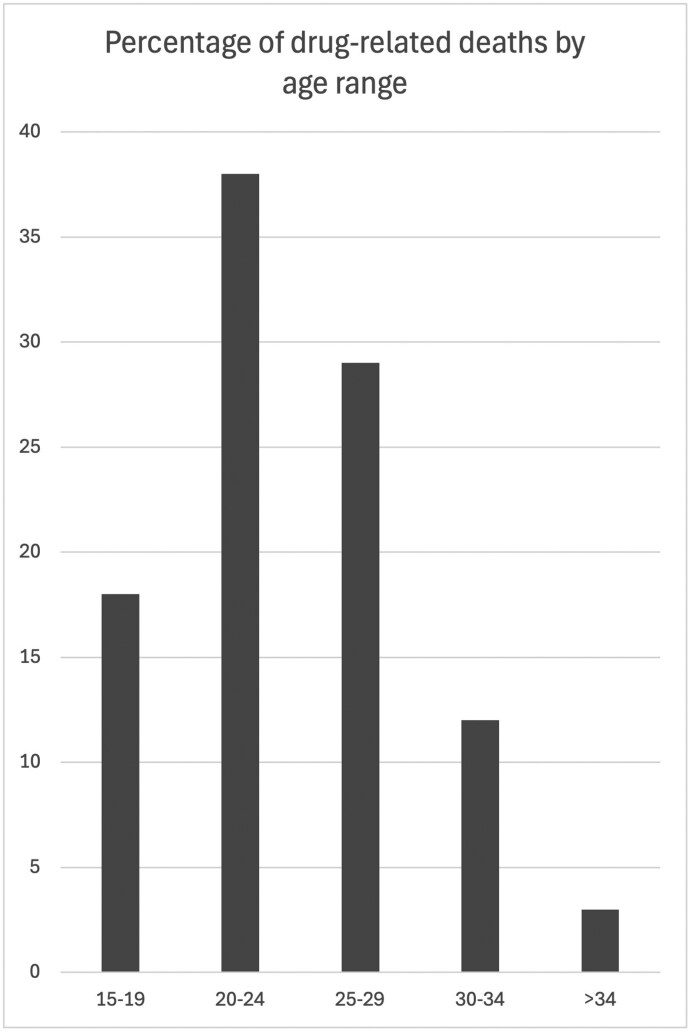
Percentage of drug-related deaths by age range, 1971–83

The picture is very different for the men who show up in the coroner’s files. Of the 34 men represented in these cases, six (18 per cent) were teenagers, aged between 15 and 19 years old, 13 (38 per cent) were in their early-twenties (20–24 years old), 10 (29 per cent) were in their mid- to late-twenties (25–29), four more (12 per cent) were in their early-thirties (30–34) and one was aged 54 (Figure 2). This final, older, man is an outlier in the set. Like the women mentioned above he had longstanding substance-dependence issues but was not connected to any social drug culture. He was a labourer who had become addicted to painkillers after an accident which left him largely confined to his bed for the rest of his life. He had started crushing and injecting his prescribed medication and contracted a fatal case of septicaemia from this practice. If we exclude this outlying case and look only at the 33 cases where the men were linked to a drug culture, we can see that the tendency towards youth is consistent across both periods, with the average age of men who died in the 1971–6 period being 22 years old, rising to just 24 years old in the later period. In spite of this slight change in the age profile of the deaths, the age at which these men started using drugs remained remarkably similar, ranging from 14 to 27 years old, but maintaining a stable average, across both periods, of a few months under 20 years old.

The picture that emerges is a remarkably clear one of a subculture of primarily working-class young men with no, or unstable, employment. When we look closer, however, a small counter-image also emerges of primarily middle-aged people, mainly women, using drugs alone and secretively. However, these homogenised images can obscure people’s lived reality. In the next two sections, I will discuss the lives and deaths of Michael Doyle and Sr Eleanor Nolan, whose stories are both representative of these two images, while also being deeply individual.

### Glimpse 1: Michael Doyle^41^

The accounts of Michael Doyle’s final day give us a glimpse into the masculine, working-class world of Dublin’s drug scene in the early-1970s. In 1972, Doyle was 27 years old, unemployed and living with his mother and younger brother in Drimnagh, a large area of 1940s and 1950s council estates on the south bank of the Grand Canal. One late January morning, Doyle arranged to meet some friends at the Labour Exchange, where they would draw their dole.^42^ After going to ‘the labour’, Michael went home while three of his friends went to town and ‘scored some “sleepers,”’ [barbiturates]. Having scored, one of the three took some with his tea and fell asleep for the evening.^43^

Throughout this article, I have intentionally included a degree of banal detail. When we, as historians, write about subcultures, like the drug scene, it is tempting to only relate the operative details—what drugs people used, how the state responded, how users interacted with services, and so on. What I hope to capture in this article is the everydayness of drug use. The cases described were not ‘drug-users’ they were ordinary people who had a few pints and went for fish and chips with their mother like so many other people, or who uneventfully fell asleep after taking sleeping pills.

That evening, at around 7.30 pm Michael and his mother went across the canal to the Bridge House Pub, in the Dolphin’s Barn area, where Michael drank four or five glasses of whiskey and four pints in just 4 h. Afterwards he and his mother, Patricia, ate a dinner of fish and chips. On their way home she noted that he did not seem drunk.^44^ Before reaching their home, Michael sent his mother on ahead while he stopped into a house a few doors up to see some friends. He seemed excited to go in and actually ran up to the front door. When he arrived there were, excluding the owner’s baby, already nine people in the small house, all aged between 17 and 23, all from the Drimnagh area, and all, except one, were men.^45^ Of the five whose occupations were recorded in the coroner’s file three were unemployed and the other two were a house painter and a fitter. Earlier that day, Jim Ryan (20 years old), who lived in the house, decided that he and his wife did not have ‘enough bread [money]’ to go to the pub so they would stay in to play cards and listen to records. As the group gradually joined them it became a bit of a party, with the friends crowding into the kitchen smoking joints and taking tablets from the two large plastic bags sitting on the table—the bags that Michael’s friends had bought earlier in the day. The coroner later noted that the bags would each have contained hundreds of pills. Even though the men were told they were Artanes, a type of anti-cholinergic which was commonly used recreationally in the early 1970s as a euphoriant and hallucinogenic, the bags contained an array of tablets and capsules of several different types. One person who was there described discussing what a safe dose was for these particular drugs, before taking five ‘Artane’ tablets. He said that by the time Michael arrived they ‘were all fairly well stoned and staggering’.^46^ When Michael joined the party, he told the group in the kitchen that he was going to ‘do himself in’ and tried, and failed, to cut his chest with a blunt bread knife. However, one witness observed, ‘nobody took him seriously as he had said that millions of times. Everyone could see he was just trying to impress us.’^47^

After this, he took a ‘fistful’ of red and blue capsules (probably the barbiturate Tuinal, which was commonly prescribed as a sedative, not Artanes). He swallowed some, and, sitting by the fire, had a friend dissolve and inject him with the others. Afterwards, he told the party that he was ‘flying’.^48^ A little later, the owner of the house and his wife went to bed, taking the bags of drugs with them because they were concerned that someone would overdose accidentally. Stumbling out of the house, a little after midnight, Michael told his friend that he wanted to go to the drug clinic in Jervis St Hospital. His friend replied ‘It’s O.K. it has happened to us all and I’ll see you tomorrow.’^49^ At home he fell asleep on the kitchen floor. It was not the first time he had fallen asleep in the kitchen after the pub, so his mother, thinking he was drunk, put a pillow under his head, wrapped him in a rug, and left him there to sleep it off. The next morning, she came downstairs to discover that he had not moved, thinking he was in a coma, she ran next door to ask her neighbour, who had a telephone, to call an ambulance. By the time the ambulance brought him to Dr Steven’s Hospital, he was recorded as ‘BID’ (Brought in Dead). His autopsy revealed that he had died from an overdose of barbiturates.

### Glimpse 2: Sr Eleanor Nolan^50^

While the case file about Michael Doyle’s death captures the drug culture at its most statistically normal, it is important to recognise that it does not capture the full picture. Although it happened less than two kilometres from where Doyle died, the case of Sister Eleanor Nolan was markedly different. Before discussing this case, however, it is important to acknowledge that we can know far less about people who used their drugs alone, or who were protected from interactions with the state by family or institutional structures. In this case, we can see that not only was Sr Eleanor using drugs alone, but there was also a firm institutional silence around where she was getting them, and how she had first started using them. To draw a blunt comparison between the available information on the cases of Sr Eleanor Nolan and Michael Doyle, we could note that Doyle coroner’s file is 35 pages long, while Sr Eleanor’s is just 12. Interestingly, this silence was maintained by the media which did not report on the inquest at all, despite regularly reporting on other drug-related deaths, at times even publishing prominent front-page articles about the evidence given in coroner’s courts.^51^

Sr Eleanor was a member of the Order of the Little Sisters of the Assumption, living in the Seven Oaks Convent on Sarsfield Road in Kilmainham. She came from a middle class area of Dublin, from a family in which all three daughters went on to become nuns.^52^ She entered the Order in 1944, aged just 18 and, after spending some time working for the Order in London, she returned to Dublin and joined the Seven Oaks Convent around the time her mother died in 1971/72.^53^ At this time she seems to have developed a dependency on opiates, but managed to keep it a secret from her friends and the rest of the community.^54^ She spent her first 6 years in the Dublin convent as a community nurse, working with families in the Kilmainham area. In 1977, she got a new job working with a child-minding agency. With this job, she would spend weeks at a time away from the convent and when she came back she ‘always appeared to be very unhappy’.^55^ After about 6 months of this, the community became aware that Sr Eleanor had ‘a drug problem’.^56^ Around this time, she began seeing a doctor to treat her substance use. On two occasions in the year leading up to her death Sr Eleanor was found by her friend, collapsed on the floor under the influence of drugs, and in early April 1979 she spent a night in Dr Steevens Hospital following an overdose.

Later in April 1979, Sr Eleanor came to the kitchen of the convent to tell the nuns working there that she would not be joining them for dinner and went straight to her room. The next morning one of the nuns went tell her that there was a phone call for her, and found her dead on the floor of her room.^57^ Near her body they found syringes containing the Palfium and Fortral, two opiates with strengths between morphine and heroin.^58^ The coroner concluded that she had died of a self-administered overdose of Palfium.^59^

### Mixtures and Changing Patterns

**Fig. 3:**
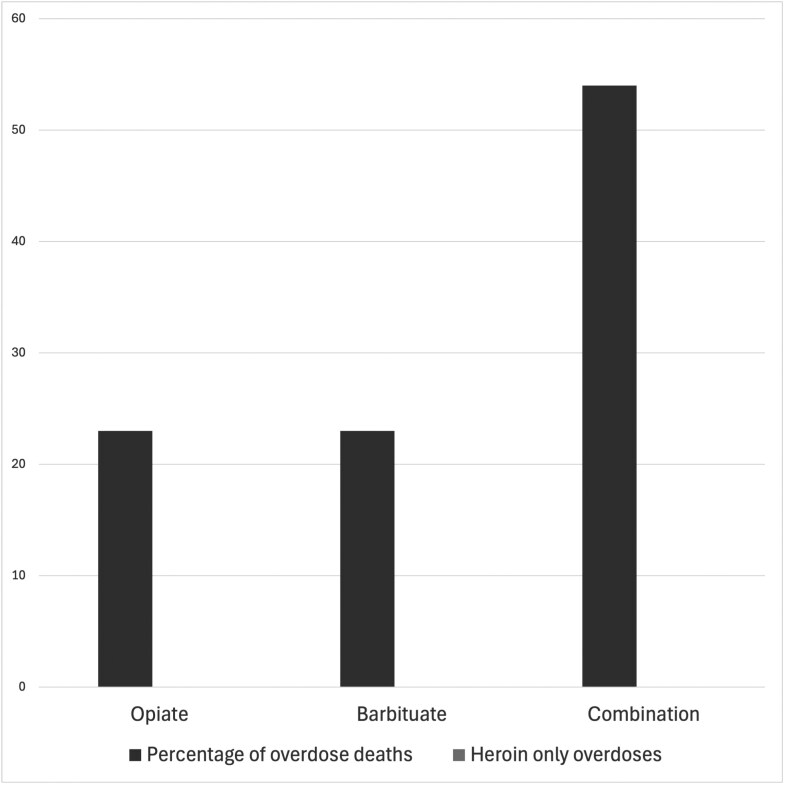
Percentage of drug-related deaths by drug implicated in the death, 1971–6

**Fig. 4:**
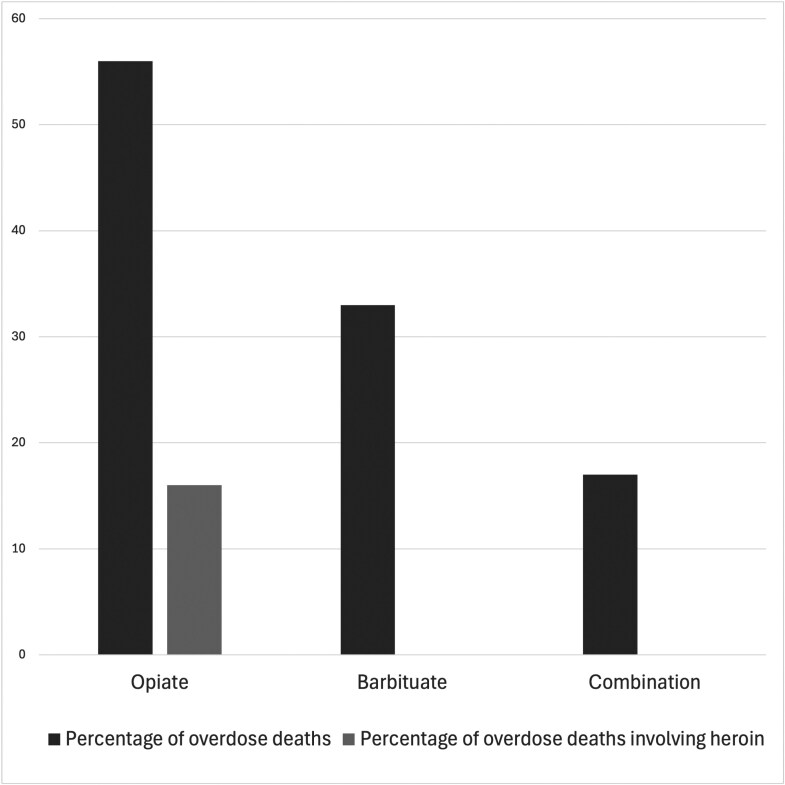
Percentage of drug-related deaths by drug implicated in the death, 1979-83

While patterns of gender, age and class remained remarkably consistent between 1972–7 and 1979–83, there were many important differences between the two periods. The most striking and significant shift was the rise in the average number of deaths recorded per year. This rose 67 per cent from 2.7 in 1971–6 to 4.4 in 1979–83. One important factor which contributed to the changing nature of the Irish drug scene was the organisation and formalisation of the supply chain for illicit drugs, particularly heroin, in 1979.^60^ Prior to this, perhaps because the market was too small, Ireland had little connection to international ‘hard’ drugs markets and the drug supply chain was chaotic. The Mullen Report made it clear that in the early-1970s drugs were mainly being procured through ‘petty thefts’, forged prescriptions and the smuggling of ‘small quantities’.^61^ The result was that users tended not to have a regular drug, but instead would use whatever was at hand, as we saw in the case of Michael Doyle who thought he was taking Artanes, but died of a barbiturate overdose. In the late 1970s, however, coherent illicit supply lines began to open up, and in July 1980 the coroner recorded the first death due to consumption of a ‘crude diamorphine preparation’, meaning illicitly produced heroin.^62^ One of the effects of these new stable supply lines is evident in the development among users of clear preferences for particular drugs. In the early 1970s, 54 per cent of deaths were linked to mixtures of different drugs (excluding mixtures of the same types of drugs, e.g., combinations of multiple opiates, and mixtures with alcohol). The remaining cases were divided evenly between deaths caused by opiates and barbiturates. By contrast in the 1979–83 period, the number of deaths attributed to mixtures of types of drugs had fallen to just 11 per cent. In the same period, the percentage of deaths caused by opiates rose to 56 per cent, and the number caused by a single, named and unmixed opiate rose to 37 per cent (Figures 3 and 4).

Given that the 1979–83 period accounts for much of what has become known as the ‘heroin epidemic’, it will come as a surprise to many that the number of coroner’s cases in which heroin was mentioned actually fell very slightly from 19 per cent in the 1971–6 period to 18 per cent in the 1979–83 period. However, in the earlier period the pharmaceutical heroin, mainly stolen from chemists’ shops, is not the immediate cause of death, while in the later period the illicitly produced heroin is. In the earlier period, there were three cases in which heroin was mentioned. In one case, a man was found drowned, and when his body was searched amounts of heroin, cocaine and morphine were found. In another case, a man died of liver failure which was attributed to hepatitis B which he probably acquired from intravenous heroin use. The third case was of a man who had previously been found in possession of heroin, and who died from an overdose of a cocktail of the milder opiate Omnopron (a morphine and codeine solution) and Antrenyl (a milder non-opiate analgesic), which he took because he could not get his preferred opiate, Physeptone (the only brand of methadone available in Ireland, it was used in opiate substitution programmes).^63^ So, in these cases, although heroin is mentioned in the coroner’s files it was unrelated to the cause of death in one case, and in the other two was only an exacerbating factor. In the later period, on the other hand, there were four files which mentioned heroin. In three cases, they were overdoses, one of heroin alone, one in combination with alcohol and one in combination with Mogadon (a benzodiazepine which was, at the time, advertised to doctors as a non-addictive tranquillizer).^64^ The fourth death was a man who had a heart attack caused by his withdrawal from heroin. Nonetheless, 18 per cent of drug-related deaths is a surprisingly small proportion, and while no single drug is mentioned more often than heroin, Palfium, another powerful opiate, is mentioned just as often.

While the types of drugs were changing, the way that they were being used also changed. Three-quarters (75 per cent) of the deaths in the 1971–6 period were linked to injected drug use. By the 1979–83 period, this had fallen below half (47 per cent). This is surprising given the cultural association between the heroin epidemic and the rise of an injecting culture. It is tempting to assume that this was because the later period included a greater number of new users, but this is not borne out by the figures. In fact, using the 21 case files that mentioned the length of time that the deceased had been using drugs before their death, we can see that the average time from first use to death actually rose from 2 years and 11 months to 4 years and 11 months. Due to the limited data available, we cannot draw reliable conclusions about the different amounts of time that the injecting and non-injecting drug users were using before their deaths. However, we can say that the distribution of new and veteran users was relatively even, ranging from 3 months to 12 years with a slight bias towards newer users—with 18 per cent under 1 year, 27 per cent at around 2 or 3 years, 23 per cent around the fourth or fifth year and the remaining deaths distributed between the sixth, eighth and twelfth years.

In the case file of Tom Plunkett, we can see how changing patterns of drug use manifested on an individual level, from the varieties of drugs that he used; the lengths he would go to get them, from doctor’s visits to pharmacy burglaries; to the ways that these efforts brought him into contact with the criminal justice and medical systems.

### Glimpse 3: Tom Plunkett^65^

In late June 1969, Tom Plunkett, then 17 years old, was playing football with the team of the factory where he worked. After the match he got into a car with his 14-year-old brother, Philip, who worked in a city-centre fur factory; Christy Kennedy (15), a sawmill worker; Henry Curley (18), unemployed; and Paul Cullen (19), unemployed. After driving into the evening, the car sped out the long straight Navan Road, pursued by a Garda squad car, while other Gardaí attempted to set up a roadblock. However, while zigzagging, presumably to prevent the squad car from overtaking, the car struck the pillar of the Clonee Bridge and flipped over seriously injuring Tom Plunket and one other boy and killing Tom’s brother Philip.^66^ After this traumatic incident Tom’s life started going off the rails. Five months later, he was sentenced to 6 months for stealing a car from Kildare St, outside the Dáil, with three friends and leading the Gardaí on a 25-min chase which ended in a crash with a stationary car in Chapelizod.^67^ Around this time, he also moved out of the family home in Ballyfermot for the first time. He would spend the next 5 years moving back and forth between ‘various flats in the city’ and his parents’ home.^68^ It was around this time that Tom began using drugs regularly.

By 1971, Tom had become well known to the Garda Drug Squad as a user of ‘all types of drugs’, and in the summer of 1973 the Gardaí noted his interests had begun to focus specifically on ‘morphine and morphine derivatives’.^69^ In November of that year, he was arrested for possession of opium and cannabis which he had hidden under the front seat of his friend’s car. While his three friends, who were also in the car, were sentenced, Tom skipped bail and absconded to the UK.^70^ As his friend Larry Quinn (24) was having his case heard at Kilmainham Court, Justice O’Sullivan said:

Get off drugs for your own sake, for your own life, not for me, not for your girl, or not for your mother. It is your life, and you’re going to be a slave to that tablet if you don’t act now […] This is not a punishment. I am doing this for your sake.

After this warning, the judge postponed his decision on the case by 3 months so that Quinn would be able to continue attending Jervis Street Hospital where he was being tapered off the drugs. Tom returned to Ireland in 1974 when he too was trying to wean himself off drugs, although without medical help. His girlfriend, Mary Fitzwilliam, recalled that by the autumn of that year, he was down to just one injection per day.^71^ Around this time, however, he was discovered by the Gardaí in a flat in the South Dublin suburb of Dun Laoghaire in possession of a ‘large amount’ of morphine, both ampules and tablets, and a ‘small quantity of heroin’.^72^ He was arrested again on 18 October with his friend Des O’Sullivan while trying to fill an apparently legally obtained prescription for Diconal. When he was arrested, Tom admitted that he had broken into four pharmacies since coming back to Ireland. The Gardaí seem to have taken a similarly paternalistic approach to Tom’s drug-use to that adopted by Justice O’Sullivan in 1973. Having admitted to the break-ins Tom asked to be taken to a doctor. The Gardaí took him to Richmond Hospital and then to the Jervis Street Drug Clinic, where he was seen by a doctor and a social worker. He wanted to be admitted to Jervis Street and given methadone. The doctor said she would arrange a bed for him in St Loman’s psychiatric hospital in County Westmeath, but while she was arranging it, Tom became restless and said he would not go. The Gardaí then took him back to Dun Laoghaire where he was charged and released on bail before the detectives dropped him and O’Sullivan to Grafton Street in the city centre.

After his release, Tom was seen on Grafton Street with some friends at 9.30 pm, and not long after that arrived at another friend’s flat on Commons Street in the inner-city docklands area, where he had been staying for the last week. Mary, Tom’s girlfriend, who was also in the flat, recalled that he was complaining of withdrawal symptoms, so he took an injection from a phial of Omnopron, a morphine and codeine solution. After this he ate dinner and, at around 11.30 pm told Mary that he was going out for a short while. At the time he was laughing and joking, suggesting he might go back to the hospital for a ‘linctus’, probably meaning Physeptone, to help with his continued withdrawal symptoms.^73^

What happened over the next 2 hours is unclear, but he arrived at a friend’s flat in Thomas Clarke Tower, 6 km away in Ballymun, at around 1.30 am ‘very much under the influence of drugs’.^74^ He had a seizure at the flat and told his friend that he had taken Antiphen, a mild paracetamol-based painkiller. Concerned that he was overdosing, Tom’s friend brought him down to the main road and tried to flag down a taxi. Several slowed but drove on when they saw the two men close up. Finally, one stopped and took Tom and his friend to Richmond Hospital. By the time they got there, at 1.55 am Tom was comatose. With the help of a porter, they carried him into the casualty department, made sure he was seen by a doctor and left. Over the next day, his friends and girlfriend visited him in hospital, but after three heart attacks, he died at 8.10 am on 20 October. His cause of death was ruled to be respiratory failure following an overdose of Antrenyl, and anti-cholinergic drugs like Atropine.

### Spatial Profile

The most revealing pieces of information that we find in the coroner’s reports are the places where people were dying from drug use. Using these reports, we can construct a geographic profile of fatal drug use in the period.^75^ In the early period of our study, 1971–6, the vast majority (81 per cent) of the deaths occurred in the city’s suburbs, with two-thirds of those occurring in the South of the city. Half (50 per cent) of the people who died lived in areas of working-class housing estates that had been largely built by local authorities in the preceding 35 years: Finagles, Drimnagh, Ballyfermot, Crumlin and Whitehall. The area which was most often given as an address for the deceased, however, was Rathmines, an area considered ‘flatland’ because of its high density of bedsits in ill-kept Victorian houses. It had a large transitory and young population.^76^

In the later period (1979–83), however, the distribution of places where people took fatal overdoses was far more widely spread across the city. The South still accounted for the greatest proportion (32 per cent), followed by the South inner city (27 per cent), the North (23 per cent) and finally the North inner city (18 per cent). This reflected a change in the communities that people were coming from. The number of people who came from council-built working-class suburbs fell from 50 per cent to 30 per cent, and while only one person had come from an inner-city area in the earlier period (North Strand), in the later period 45 per cent came from the inner city. The Northern suburb of Ballymun and the South inner-city area of Rialto were particularly hard hit, and each accounted for 15 per cent of the deaths. In Rialto, this figure doubled to 30 per cent if we take into account nearby deaths in the contiguous communities of Drimnagh, Inchicore and Kilmainham.

That drugs took root in different communities only forms part of the picture about the widening distribution of places where overdoses happened. It is also indicative of a change in the kind of places where drugs are being used. In the earlier period, 1971–6, 81 per cent of fatal incidents occurred in private homes, and more than three-quarters of those (63 per cent of the total) occurred in the deceased’s own home. In 56 per cent of cases the deceased lived at home with parents and siblings, and in 31 per cent of cases the fatality happened in that family home. Over the decade the number of deaths happening in homes fell drastically, and in the later period, only 50 per cent of incidents were occurring in private homes, just 41 per cent happening in the deceased’s own home. In this period, the number of people who had incidents and lived with their relations also fell to 45 per cent, and the number of fatal incidents occurring in these family homes fell to 18 per cent. In the earlier period, only one overdose death occurred in a public space, Bushey Park in Terenure South Dublin. This changed drastically in the later period when 41 per cent of overdoses occurred in public spaces. The location and types of these spaces varied and included three pubs, a city-centre pizza restaurant, on two street corners, in a public park and the communal basement area of a large block of flats.

In the case of Gerry O’Neill, we can see what it meant, on an individual level, to use in different places, from public city-centre places to private homes in the suburbs.

### Glimpse 4: Gerry O’Neill^77^

On the afternoon of his 21st birthday, Gerry O’Neill left his home in Fatima Mansions, in the South inner city. He met a friend, Patrick O’Connor (27) whom he had known for about 6 months, and together they went to Stephen’s Green. When Patrick discovered that it was Gerry’s birthday, he ran back to his flat, around the corner on Cuffe Street, to get a bottle of whiskey and a ‘naggin’ [small flask] of gin. The two sat down with their drinks in the park near the statue of W.B. Yeats. After a little while a stranger approached them, he shared some of his Valium with them and they shared their drink with him.^78^ After a while Albert Casey (33) arrived and Patrick took out ‘a bit of draw’ [cannabis], and they smoked a few joints. By 6.15 pm, they were all a bit stoned, and Albert told the other two that they could go back to his house, while he went off for a few pints in The Sword pub on Camden Street. Gerry and Patrick decided to ‘score some smack’.^79^ They got a taxi to Donore Avenue, near Gerry’s home, in the South inner city. Gerry said he knew where to get ‘some good gear’, so Patrick waited in the car while Gerry scored. He came back with three £10 packs of heroin, and they then went back to Albert’s house in Hillview Estate, a new working-class estate in Ballinteer at the Southern edge of the city.

When Gerry and Patrick got to the house, they found that the only people there were Albert’s four children—his wife being in hospital with an infection in her foot. The two men left the children to play downstairs and went to an upstairs bedroom where they discussed how they should use the heroin:

I had asked Gerry if he would prefer to snort or skin pop [subcutaneously inject] the heroin but he insisted on mainlining [intravenously injecting] and said he was well used to it and had used that particular heroin before.^80^

They took turns to inject each other, and Patrick recalled that ‘about fifteen minutes after Gerry had his fix he fell into my arms’.^81^ He put Gerry in an armchair and poured a jug of cold water over him to revive him, but it didn’t work so he gave him to ‘kiss of life’, mouth-to-mouth resuscitation. When he revived Patrick took off his wet shirt and put a blanket around him before going down to check on the children who were alarmed by the commotion upstairs and needed to be calmed down.^82^ At around midnight Albert came home ‘a bit gargled’ [drunk] to find Gerry and Patrick ‘stoned out of their heads’.^83^ Gerry was obviously more stoned, and Albert was worried about him, so he dragged him downstairs and slapped him until he started muttering. This reassured Albert that Gerry was alive and comparatively safe, so he put him to bed under an overcoat on the living-room floor.

When Albert awoke at 7 am he remembered what happened to Gerry and went straight down to check on him. He could not wake him but thought he could feel a faint pulse. He woke Patrick and ran to the house of his neighbour Robert Kavanagh (26), who had a van, saying ‘there’s a guy over here and he is after O.D.’n’. By the time Robert got to Albert’s house, Gerry was cold and ‘looked like a corpse’.^84^ They took him to St Vincent’s Hospital, but the hospital recorded him as ‘Admitted BID’.^85^

The coroner found that his death was due to misadventure, caused by an overdose of alcohol and drugs, taken in both Stephens Green and in the house in Ballinteer. Within the pathologist’s report, however, there are some interesting details about the syringe that they used. The first thing is that there was only one syringe, implying that they were sharing their work. Next the pathologist remarks on the fact that it was a plastic 2-ml syringe intended to be used once and thrown away, but it was scuffed as though it had been carried around in a pocket or bag, and the residue inside suggested it had been used multiple times. Moreover, there were residues of at least two different drugs in the syringe. There was a pinkish white powder caught around the plunger end which the pathologist suggests was the residual trace of an earlier use. This powder included dipipanone and was probably the remains of Diconal which came in pink tablets. The other was the remains of a white powder found primarily at the needle end and was probably the residue of the most recent use of the syringe. Tests revealed that this included morphine, monoacetylmorphine and diamorphine. The forensic scientist noted that ‘diamorphine (heroin) is produced by the acetylation of morphine, monoacetylmorphine being formed during the process.’^86^ This is consistent with what the forensic scientist called a ‘crude diamorphine preparation’, that is to say illicitly produced heroin.^87^ This is the first reference in any coroner’s report to illicitly produced heroin, as opposed to the ampules of pharmaceutical heroin stolen from chemists that had previously been in circulation.

## Conclusion

This article set out to explore ‘round all the sides’ of the first 12 years of the hard drug scene in Dublin.^88^ In doing so, it challenged the perception that the hard drug scene began in Ireland at the end of the 1970s, driven by singular causes like the Iranian revolution and a particular gang’s decision to start importing drugs. While these played an important role in formalising the drug supply, and as a result shaped the culture, this article has shown that the ‘hard drug’ scene predated these events by about a decade. The article aggregated the data from coroner’s files to create an image of the drug culture, assessing the consistency of the age, class and gender profiles, as well as the changing nature of drug use and the evolving geographical profile.

Through this work, we could construct an agglomerate image of an ‘average’ person who died of drug-related causes in this period. Every detail of the following description is the most statistically likely, based on the data in the coroner’s reports. He was a man in his early- to mid-twenties, from a local authority-built working-class suburb of Dublin. In the early-1970s, he probably lived there with his parents, while in the early-1980s he was probably living independently, though he was unemployed. He started using drugs when he was a few months shy of 20 years old and continued to use them until his death 2–4 years later. In the early 1970s, he used a mixture of pharmaceuticals which he either stole from pharmacies or bought directly from the people who stole them. He then ground up or dissolved these ‘smarties’ so that he could inject them in the privacy of his own home.^89^ By the early 1980s, he preferred to just use opiates which he bought from drug pushers with relatively stable supply lines. He was less inclined to inject his drugs, and he was almost as likely to use in a public space, like a pub or park, as in a private home. In both periods he died alone of an accidental overdose.

We can also construct a less distinct counter-image of the average woman who died from drug-related causes, who presented a very different profile to the men. She was 48 years old and was equally likely to be from a professional background or to be a working-class housewife. She had been using drugs for 8 years and kept it secret from her friends and family, though her doctor knew about the habitual use and may have provided some or all of the drugs she used. She lived alone and died from an overdose of barbiturates taken in her own bedroom.

These agglomerations are the equivalent of the early-modern Wound Man, illustrative, certainly and useful in their own way, but not representative of any lived reality. To counteract this impersonality, each discussion of a statistical trend has been accompanied by one or two cases that demonstrate how the trend actually played out in people’s everyday lives and deaths. I have tried to stress the ordinariness of these stories—Michael Doyle having fish and chips with his mother, Tom Plunkett playing football with his workmates and Gerry O’Niell’s friend running back to his flat to get a naggin of gin to toast Gerry’s birthday.

While the article has captured this ordinariness, what it lacks is the everyday lived experience of love and joy. This is obscured by our cultural preconceptions about hard drug scenes, particularly in working-class contexts, but more than that it is obliterated by the inevitable tragedy of the source material. The fatal context provided by these reports turns moments of everyday love, like Michael Doyle’s mother putting a pillow under his head and wrapping him in a blanket, into tableaux of tragedy. In light of this, I hope that this article will serve three purposes. Firstly, as an insight into the patterns and trends of the first decade of the hard drug scene in Dublin. Secondly, as a glimpse into the ordinariness of the drug culture and an illustration of how it existed within a wider social continuum, rather than as a separate underworld distinct from the rest of society. Finally, I hope that the article will invite further study into the history of emotions within drug cultures, and, in particular, the neglected experience of love and joy.

## Footnotes

1 Niall O’Dowd, ‘Dublin “Is the Heroin Capital of Europe,”’ *Irish Press*, 22 June 1983, 3.

2 Irish Medical Journal, March 1968.

3 This narrative was established as early as 1983 through articles like Mary Raftery, ‘The Dunnes: The Inside Story of a Criminal Family’, *Magill*, 31 October 1983.

4 I have discussed this ‘Irish exceptionalism’ in relation to drugs history with James Grannell in Oisín Wall and James P. Grannell, ‘From “Opium Smoking Orgies” to “Junkie Babies”: Representations of Heroin-Use in Ireland, 1915-90’, *Cultural and Social History*, 2023, 21, 2, 95–116.

5 Ciara Breathnach, *Ordinary Lives, Deaths, & Social Class: Dublin Coroners Court, 1876-1902* (Oxford: Oxford University Press, 2022), 2.

6 Edwin Abbott, *Flatland: A Romance of Many Dimensions* (Project Gutenberg, 2022), 45.

7 Oisín Wall and James P. Grannell, ‘From “Opium Smoking Orgies” to “Junkie Babies”: Representations of Heroin-Use in Ireland, 1915-90’, *Cultural and Social History*, 2023, 20, 1, 95–116, 6–9.

8 Jo Murphy-Lawless, *Fighting Back: Women and the Impact of Drug Abuse on Families and Communities* (Dublin: The Liffey Press, 2002); Julie O’Toole, *Heroin: A True Story of Drug Addiction, Hope and Triumph* (Dublin: Maverick House, 2005); André Lyder, *Pushers Out: The Inside Story of Dublin’s Anti-Drugs Movement* (Victoria: Trafford, 2005).

9 Janet Weston and Virginia Berridge, ‘AIDS Inside and Out: HIV/AIDS and Penal Policy in Ireland and England & Wales in the 1980s and 1990s’, *Social History of Medicine*, 2018, 33, 1, 247–67; Shane Butler, *Alcohol, Drugs and Health Promotion in Modern Ireland* (Dublin: Institute of Public Administration, 2002), 134–208; Aileen O’Gorman, ‘Illicit Drug-Use in Ireland: An Overview of the Problem and Policy Responses’, *Journal of Drug Issues*, 1998, 28, 1, 155–66.

10 Stuart Hall and Tony Jefferson (eds), *Resistance through Rituals: Youth Subcultures in Post-War Britain* (London: Routledge, 2006), 4.

11
*Ibid.*, 7.

12
*Ibid*.

13 Oisín Wall and James P. Grannell, ‘From “Opium Smoking Orgies” to “Junkie Babies”: Representations of Heroin-Use in Ireland, 1915-90’, *Cultural and Social History*, 2023, 20, 1, 95–116.

14 André Lyder, *Pushers Out: The Inside Story of Dublin’s Anti-Drugs Movement* (Bloomington: Trafford, 2005), 16–25.

15 Anon, ‘Killer Disease Is Here’, *Irish Press*, 13 May 1983, 7; Anon, ‘Killer Disease Is Here’, *Cork Examiner*, 11 May 1983, 10.

16 Dermot Walsh, *Suicide, Attempted Suicide and Prevention in Ireland and Elsewhere* (Dublin: Health Research Board, 2008), 24.

17 The National Archives of Ireland reference numbers were: CC/Coroners Inquest/1971/62; CC/Coroners Inquest/1971/231; CC/Coroners Inquest/1971/263; CC/Coroners Inquest/1972/28; CC/Coroners Inquest/1972/29; CC/Coroners Inquest/1972/190; CC/Coroners Inquest/1973/37; CC/Coroners Inquest/1973/36; CC/Coroners Inquest/1973/34; CC/Coroners Inquest/1974/225; CC/Coroners Inquest/1974/274; CC/Coroners Inquest/1974/290; CC/Coroners Inquest/1974/354; CC/Coroners Inquest/1975/136; CC/Coroners Inquest/1976/150; CC/Coroners Inquest/1976/170; CC/Coroners Inquest/1976/205; CC/Coroners Inquest/1979/129; CC/Coroners Inquest/1979/295; CC/Coroners Inquest/1979/166; CC/Coroners Inquest/1980/18; CC/Coroners Inquest/1980/1; CC/Coroners Inquest/1980/41; CC/Coroners Inquest/1980/39; CC/Coroners Inquest/1980/112; CC/Coroners Inquest/1980/178; CC/Coroners Inquest/1980/152; CC/Coroners Inquest/1980/314; CC/Coroners Inquest/1980/377; CC/Coroners Inquest/1981/219; CC/Coroners Inquest/1981/186; CC/Coroners Inquest/1981/301; CC/Coroners Inquest/1982/189; CC/Coroners Inquest/1982/315; CC/Coroners Inquest/1982/375; CC/Coroners Inquest/1983/14; CC/Coroners Inquest/1983/89; CC/Coroners Inquest/1983/151; CC/Coroners Inquest/1983/153; CC/Coroners Inquest/1983/217; CC/Coroners Inquest/1983/225.

18 National Archives of Ireland, CC/Coroners Inquest/1981/301.

19 John Murphy, Statement of John Murphy, In National Archives of Ireland, CC/Coroners Inquest/1981/301.

20 Camillus Fitzpatrick, Statement of Garda Camillus Fitzpatrick, In National Archives of Ireland, CC/Coroners Inquest/1981/301.

21
*Ibid.*; Derek Connolly, Statement of Derek Connolly, In National Archives of Ireland, CC/Coroners Inquest/1981/301.

22 John Murphy, Statement of John Murphy, In National Archives of Ireland, CC/Coroners Inquest/1981/301.

23 Ellen Connolly, Statement of Ellen Connolly, In National Archives of Ireland, CC/Coroners Inquest/1981/301.

24 When referring to opioids in this article, I have given vague indications of relative strength, using morphine and heroin as commonly understood points of reference. To do this, I have drawn up opioid conversion charts that are commonly available through healthcare providers. However, these relative strengths are, at best, approximate indicators. The drugs in questions were circulating illicitly and we cannot know whether they had been adulterated, or how their strength may have been altered.

25 John Murphy, Statement of John Murphy, In National Archives of Ireland, CC/Coroners Inquest/1981/301.

26 Anne Whelan, Statement of Anne Whelan, In National Archives of Ireland, CC/Coroners Inquest/1981/301.

27
*Ibid*.

28 John Murphy, Statement of John Murphy, In National Archives of Ireland, CC/Coroners Inquest/1981/301; Anne Whelan, Statement of Anne Whelan, In National Archives of Ireland, CC/Coroners Inquest/1981/301.

29 Anne Whelan, Statement of Anne Whelan, In National Archives of Ireland, CC/Coroners Inquest/1981/301.

30 Ellen Connolly, Statement of Ellen Connolly, In National Archives of Ireland, CC/Coroners Inquest/1981/301.

31 Dick Connolly, Statement of Dick Connolly, In National Archives of Ireland, CC/Coroners Inquest/1981/301.

32 Anne Whelan, Statement of Anne Whelan, In National Archives of Ireland, CC/Coroners Inquest/1981/301.

33 John Murphy, Statement of John Murphy, In National Archives of Ireland, CC/Coroners Inquest/1981/301.

34 Anon, ‘Family Protest at Drug Inquest’, *Irish Press*, 5 December 1981, 7.

35 James Connell, Letter from James Connell to Dr Bofin, In National Archives of Ireland, CC/Coroners Inquest/1981/301.

36 Anne Whelan, Statement of Anne Whelan, In National Archives of Ireland, CC/Coroners Inquest/1981/301.

37 Niall Gallagher, Deposition of Dr Gallagher, In National Archives of Ireland, CC/Coroners Inquest/1981/301; Anon, ‘Drug addict “beaten in station,”’ *Evening Press*, 4 December 1981, 9.

38 Patrick Bofin, Record of Verdict, In National Archives of Ireland, CC/Coroners Inquest/1981/301.

39 Anon, ‘Family Protest at Drug Inquest’, *Irish Press*, 5 December 1981, 7.

40 This information is not always available. For instance, the reports only recorded the address of the family of origin in 74 per cent of cases. However, of these 70 per cent of cases come from Dublin working class communities while a further 14 per cent came from mixed communities. But in almost all cases there was at least one other marker, other than occupation which indicated a class position.

41 National Archives of Ireland, CC/Coroners Inquests 1972/29.

42 Eddie Smith, Statement of Eddie Smith 31/1/1972, In National Archives of Ireland, CC/Coroners Inquests 1972/29.

43 It was later suggested in the Coroner’s Court that the drugs had been stolen from the nearby Convent of the Little Sisters of the Poor in Kilmainham—I have not been able to find any record of prosecutions linked to this theft, however, so it must remain one of the inevitable ambiguities produced by the interaction of state power and a criminalised subculture. Anon, ‘Inquest Told How Man Died after Drugs Party’, *Irish Independent*, 5 February 1972, 5; John Peterson, Statement of John Peterson 5/2/1972, In National Archives of Ireland, CC/Coroners Inquests 1972/29; Eddie Smith, Statement of Eddie Smith 31/1/1972, In National Archives of Ireland, CC/Coroners Inquests 1972/29.

44 Patricia Doyle, Statement of Patricia Doyle 30/1/1972, In National Archives of Ireland, CC/ Coroners Inquests 1972/29.

45 James O’Brien, Statement of James O’Brien 3/2/1972, In National Archives of Ireland, CC/Coroners Inquests 1972/29.

46
*Ibid.*

47
*Ibid.*

48 Martin Byrne, Statement of Martin Byrne 5/2/1972, In National Archives of Ireland, CC/Coroners Inquests 1972/29.

49 James O’Brien, Statement of James O’Brien 3/2/1972, In National Archives of Ireland, CC/Coroners Inquests 1972/29.

50 National Archives of Ireland, CC/Coroners Inquest/1979/166.

51 For instance, a year earlier the *Evening Press* had published a large front-page story on a case in the Dublin County Coroner’s Court. Anon, ‘Coroners Give Drugs to Addicts’, *Evening Press*, 12 April 1978, 1.

52 Anon, Deaths, *Irish Independent*, 4 October 1963, 26.

53 Anon, Acknowledgements, *Irish Independent*, 29 December 1972, 17.

54 A. P. McNally, Report to Coroner, In National Archives of Ireland, CC/Coroners Inquest/1979/166.

55 Sr Brigid Moore, Statement on Brigid Moore, In National Archives of Ireland, CC/Coroners Inquest/1979/166.

56
*Ibid.*

57 Sr Margaret Kelly, Statement on Margaret Kelly, In National Archives of Ireland, CC/Coroners Inquest/1979/166.

58 G. D. Doyle, Clinical Toxicology Report, In National Archives of Ireland, CC/Coroners Inquest/1979/166.

59 P. J. Bofin, Record of Verdict, In National Archives of Ireland, CC/Coroners Inquest/1979/166.

60 Johnny Connolly and Andrew Percy, ‘Illicit Drugs, Criminal Justice and Harm Reduction’, in Johnny Connolly and Andrew Percy, eds, *The Routledge Handbook of Irish Criminology* (London: Routledge, 2015), 416–33, 418.

61 Karl Mullen, *Report of the Working Party on Drug* Abuse (Dublin: Stationary Office, 1971), 22–23.

62 Daniel O’Driscoll, Forensic Science Report, In National Archives of Ireland, CC/Coroners Inquest/1980/314.

63 Andrew Preston and Sile O’Connor, *The Methadone Handbook: Special Edition for Ireland* (Dorset: Island Press, 1998), 3.

64 P. C. Misra, ‘Nitrazepam (Mogadon) Dependence’, *British Journal of Psychiatry*, 1975, 126, 81–2, 81.

65 National Archives of Ireland, CC/Coroners Inquests 1974/354.

66 Anon, ‘Midnight Trip Ends in Tragedy’, *Evening Herald*, 28 June 1969, 1.

67 Anon, ‘Crash Ends Ballyfermot Car Chase’, *Evening Herald*, 2 December 1969, 3.

68 Denis Plunkett, Deposition of Denis Plunkett, In National Archives of Ireland, CC/Coroners Inquests 1974/354.

69 Denis Mullins, Statement of evidence of D/Sergeant Denis Mullins, In National Archives of Ireland, CC/Coroners Inquests 1974/354.

70 Anon, ‘Youths Remanded on Drugs Charges’, *Evening Herald*, 26 November 1973, 3; Anon, ‘Three on Charge of Possessing Dangerous Drugs’, *Irish Independent*, 15 January 1974, 18.

71 Mary Fitzwilliam, Statement of Mary Fitzwilliam, In National Archives of Ireland, CC/Coroners Inquests 1974/354.

72 Denis Mullins, Statement of evidence of D/Sergeant Denis Mullins, In National Archives of Ireland, CC/Coroners Inquests 1974/354.

73 Des O’Sullivan, Statement of Des O’Sullivan, In National Archives of Ireland, CC/Coroners Inquests 1974/354.

74 Oscar Ryan, Statement of Oscar Ryan, In National Archives of Ireland, CC/Coroners Inquests 1974/354.

75 For the purposes of this profile, I will divide the city into four sections: North inner city, South inner city, North and South. The inner city as the area of the city bounded by the Grand Canal in the South, the Inchicore Railway Works and Phoenix Park in the West, the North Circular Road in the Northwest, the Royal Canal in the North and Dublin Port in the Northeast. The North and South inner city are divided by the River Liffey. Similarly, the North and South are the area north and south of the Liffey, outside of the inner city.

76 Marianne Heron, ‘Helping Out in Flatland’, *Irish Independent*, 20 March 1973, 10.

77 National Archives of Ireland, CC/Coroners Inquest/1980/314.

78 Patrick O’Connor, Statement of Patrick O’Connor, In National Archives of Ireland, CC/Coroners Inquest/1980/314.

79 Albert Casey, Statement of Albert Casey, In National Archives of Ireland, CC/Coroners Inquest/1980/314.

80 Patrick O’Connor, Statement of Patrick O’Connor, In National Archives of Ireland, CC/Coroners Inquest/1980/314.

81
*Ibid.*

82
*Ibid*.

83
*Ibid*.

84 Robert Kavanagh, Statement of Robert Kavanagh, In National Archives of Ireland, CC/Coroners Inquest/1980/314.

85 John Dinn, Autopsy Report, In National Archives of Ireland, CC/Coroners Inquest/1980/314.

86 Daniel O’Driscoll, Forensic Science Report, In National Archives of Ireland, CC/Coroners Inquest/1980/314.

87
*Ibid*.

88 Edwin Abbott, *Flatland: A Romance of Many Dimensions* (Project Gutenberg, 2022), 45.

89 Gene Kerrigan, ‘Special Feature: A Necessary Respect—The Small Legend of Karl Crawley’, *Magill*, 31 May 1982.

## Notes

Content note: This article contains discussions of substance use, death, violence, criminal activity, and suicide.

## Notes

Dr Oisín Wall is a lecturer in the School of History and the Radical Humanities Laboratory at University College Cork. His research focuses on the history of marginalised communities in modern Ireland, and he currently holds a Wellcome Trust Fellowship for the project ‘We are the heroin capital of Europe’: Marginal Communities, Health, Identity, and the Opioid Epidemic in Twentieth Century Ireland. In 2024, he published his second monograph *Prisoners’ Bodies: Activism, Health, and the Prisoners’ Rights Movement in Ireland, 1972–1985* (McGill-Queens University Press: 2024).

